# Influence of stenosis between left anterior descending and diagonal arteries on long-term patency and ischemic events after coronary bypass surgery

**DOI:** 10.1016/j.xjon.2026.101837

**Published:** 2026-05-04

**Authors:** Akinobu Ohtani, Shin Yajima, Daisuke Yoshioka, Takuji Kawamura, Ai Kawamura, Yusuke Misumi, Shinya Tajima, Masaru Matsuda, Shunsuke Saito, Shigeru Miyagawa

**Affiliations:** Department of Cardiovascular Surgery, The University of Osaka, Suita, Osaka, Japan

**Keywords:** competitive flow, coronary artery bypass grafting, coronary stenosis, graft patency, internal thoracic artery, intervening left anterior descending artery stenosis, sequential grafting

## Abstract

**Objective:**

The study objective was to evaluate stenosis severity influence in the intervening left anterior descending artery, between the internal thoracic artery anastomosis and the diagonal branch bifurcation, on internal thoracic artery left anterior descending graft events in patients undergoing coronary artery bypass grafting.

**Methods:**

We analyzed 496 patients undergoing primary coronary artery bypass grafting with internal thoracic artery left anterior descending and diagonal grafts. Stratified analysis was based on intervening left anterior descending artery stenosis severity (nonsevere <90%, n = 161; severe ≥90%, n = 335). The primary end point was freedom from internal thoracic artery left anterior descending events (composite of myocardial infarction, reintervention, and occlusion/string sign). Median follow-up was 6.8 years.

**Results:**

The nonsevere group was younger, with more left main disease, but fewer sequential grafts (*P* = .002, .035, .008, respectively). Freedom from internal thoracic artery left anterior descending events at 1, 5, and 10 years was lower in the nonsevere group (94.3% ± 1.8%, 92.9% ± 2.1%, 89.5% ± 2.8%, respectively) versus the severe group (99.1% ± 0.5%, 97.2% ± 1.0%, 97.2% ± 1.0%, respectively; log-rank *P* = .002). Proximal left anterior descending and diagonal stenoses did not affect internal thoracic artery left anterior descending events (log-rank *P* = .399, .391, respectively). Sequential diagonal grafting reduced internal thoracic artery left anterior descending events compared with individual grafting (log-rank *P* = .005). Multivariable analysis identified nonsevere intervening left anterior descending artery stenosis (hazard ratio, 2.59; 95% CI, 1.07-6.30; *P* = .035) and individual diagonal grafting (hazard ratio, 2.57; 95% CI, 1.04-6.36; *P* = .041) as independent risk factors, whereas higher internal thoracic artery left anterior descending flow (hazard ratio, 0.94; 95% CI, 0.90-0.98; *P* = .006) was a protective factor.

**Conclusions:**

Nonsevere intervening left anterior descending artery stenosis independently predicted internal thoracic artery left anterior descending events. Sequential diagonal grafting significantly mitigated this risk. Thus, a grafting strategy considering intervening left anterior descending artery stenosis is essential to ensure long-term internal thoracic artery left anterior descending patency.

## Graphical Abstract

**Figure:**
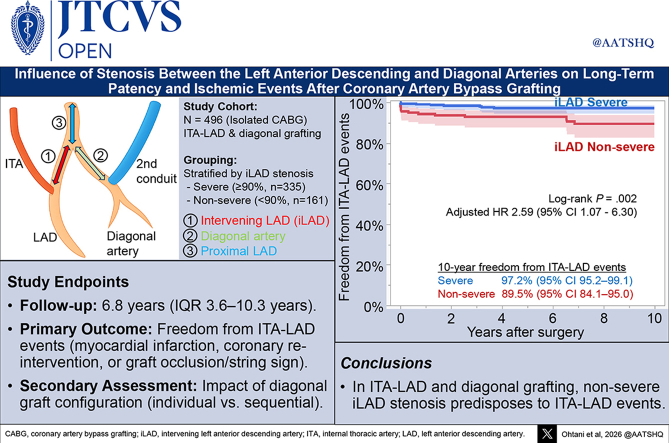

**Figure:**
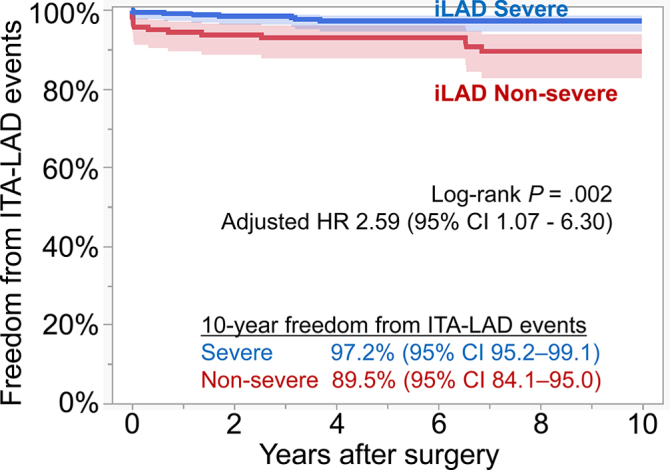
iLAD stenosis severity is a significant predictor of ITA-LAD graft failure.

**Central Message**

In ITA-LAD and diagonal grafting, nonsevere iLAD stenosis predicts ITA-LAD failure. iLAD-tailored strategy, including sequential diagonal grafting, is essential to prevent long-term ITA-LAD events.

**Perspective**

Although simulations suggested competitive flow risks in ITA-LAD and diagonal grafting, clinical evidence regarding specific predictors is lacking. This study identifies nonsevere iLAD stenosis as the dominant cause of ITA-LAD failure. Consequently, tailoring the graft strategy to iLAD severity is essential to ensure long-term ITA-LAD patency while achieving complete revascularization.

Coronary artery bypass grafting (CABG) using the internal thoracic artery (ITA) to left anterior descending (LAD) artery graft is the gold standard for managing complex coronary disease.^1^^,^^2^ Significant mid- to long-term challenge in CABG is graft atrophy (string sign), where occlusion results from flow competition between the native coronary artery and the graft, or within the graft system.^3^^,^^4^ Bypass flow to the diagonal territory may compromise ITA-LAD graft patency. Concomitant diagonal grafting is an independent risk factor for ITA-LAD failure,^5^ particularly with moderate LAD stenosis. Furthermore, a patent saphenous vein graft (SVG) to the diagonal or circumflex arteries is associated with reduced long-term ITA-LAD patency.^6^ Likewise, high blood flow from a diagonal conduit (eg, SVG) induces the ITA string sign or functional occlusion if no hemodynamically significant lesion exists between the ITA-LAD and diagonal anastomoses.^7^^,^^8^ Stenosis severity in segment between the diagonal and ITA-LAD anastomoses plays a decisive role in ITA-LAD graft hemodynamics. However, this specific segment lacks detailed investigation. To provide a comprehensive analysis of the competitive flow risk to the ITA-LAD graft, we evaluated stenosis severity influence on the proximal LAD (pLAD), diagonal artery, and the intervening LAD (iLAD), defined as the segment between the diagonal and ITA anastomoses.

## Materials and Methods

### Study Cohort

**Figure E1:**
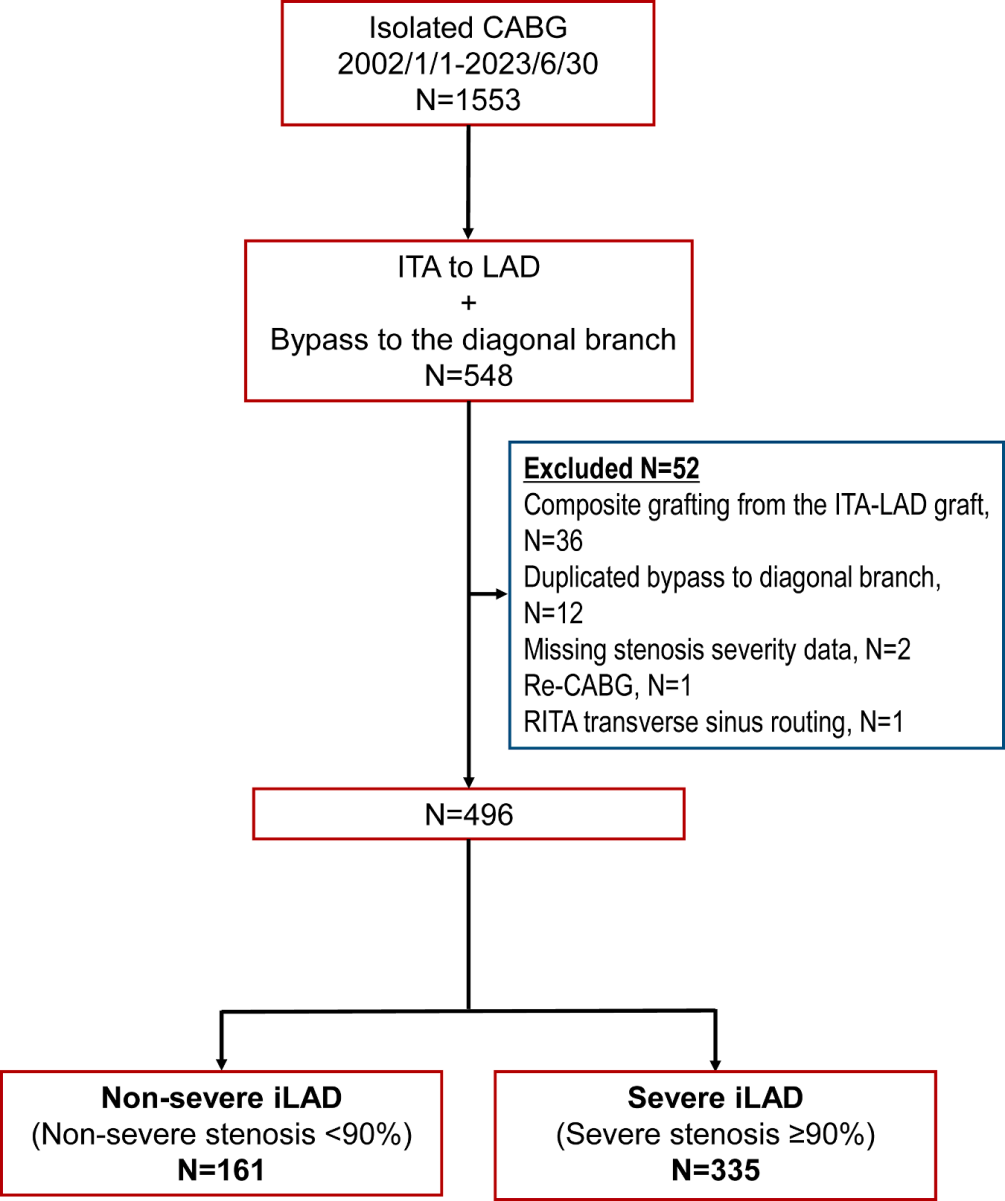
Patient flow diagram. *CABG*, Coronary artery bypass grafting; *iLAD*, intervening left anterior descending artery; *ITA*, internal thoracic artery; *LAD*, left anterior descending; *RITA*, right internal thoracic artery.

Patient selection is shown in Figure E1. This single-center retrospective study included consecutive patients who underwent isolated primary CABG at our institution between January 2002 and June 2023. Of 1553 patients screened, 548 received an ITA graft to the LAD and a diagonal graft. Exclusion included composite grafting with an ITA-LAD graft (n = 36) or re-CABG (n = 1), or duplicated bypass to the diagonal artery (use of multiple independent grafts to different diagonal branches in a single patient; n = 12) to eliminate the confounding effects of multisource hemodynamic interference and isolate single inflow source impact. Additionally, patients with missing data on stenosis severity (n = 2) or right ITA transverse sinus routing (n = 1) were excluded.

### Definitions and Graft Configurations

To evaluate stenosis location impact, stenosis severity was assessed in 3 specific segments relative to the target bypassed diagonal artery closest to the ITA-LAD anastomosis: iLAD, bypassed diagonal artery, and pLAD, which is defined as the LAD segment proximal to the diagonal artery origin, including the left main trunk. Stenosis severity was classified as severe (≥90%) or nonsevere (<90%) based on preoperative angiography findings. This classification was applied to all segments, including the left main trunk, to evaluate the risk of flow competition affecting graft patency, which is distinct from the standard clinical threshold for ischemia. Although a 70% stenosis typically induces ischemia, previous computational and physiological studies suggest that a native stenosis of approximately 90% or greater is required to create a sufficient pressure drop to neutralize competitive flow and ensure predominant forward flow from the graft.^9^^,^^10^ Therefore, the 90% threshold was strictly applied to define the conditions minimizing competitive flow risk.

Diagonal graft configuration was classified as individual (graft supplying only the diagonal territory without extending to other territories) and sequential (graft supplying the diagonal artery and extending to the lateral or inferior territory). As an institutional policy, an independent ITA graft to the LAD was prioritized to ensure its long-term patency. Sequential grafting of the ITA to the diagonal and LAD was generally avoided to prevent flow competition arising from differing degrees of native stenosis. The choice of the second conduit for the diagonal branch was based on the patient's characteristics and the surgeon's discretion.

### Intraoperative Measurements

Intraoperative graft flow was assessed using transit-time flowmetry for both ITA and diagonal grafts before chest closure under stable hemodynamics.

### Study End Points

The primary end point was freedom from ITA-LAD events, defined as a composite of any of the following adverse events related to the LAD territory or ITA-LAD graft: myocardial infarction, coronary reintervention, or graft occlusion/string sign detected on postoperative coronary computed tomography angiography or coronary angiography.

### Follow-up

After hospital discharge, patients attended outpatient visits at 1, 3, 6, and 12 months, and annually thereafter. Follow-up data were obtained from medical records and supplemented by telephone or postal correspondence. Early postoperative coronary angiography was routinely performed before discharge to confirm baseline graft patency, except in patients with contraindications to contrast media, such as allergic reactions or advanced renal insufficiency. In contrast, late follow-up coronary angiography was not protocolized but strictly clinically driven, performed only when patients developed recurrent ischemic symptoms or objective signs of ischemia on noninvasive examinations.

### Statistical Analysis

Continuous variables are presented as mean ± SD or median (interquartile range [IQR]) and were compared using the Student *t* test or the Mann–Whitney *U* test. Categorical variables are presented as numbers and percentages and were compared using the chi-square or Fisher exact tests. Long-term outcomes, including overall survival and freedom from ITA-LAD events, were analyzed using the Kaplan–Meier method. To avoid overinterpretation of sparse late data, all time-to-event analyses and corresponding survival curves were truncated at 10 years. Differences between groups were assessed using the log-rank test. Univariate and multivariate Cox proportional hazards regression analyses were performed to identify predictors of ITA-LAD events. Variables with a *P* value less than .10 in the univariate analysis were entered into the multivariate model. Statistical analyses were performed using JMP, Version 18 (SAS Institute Inc).

### Ethics Statement

This study was conducted as per Declaration of Helsinki principles. Patient data were obtained from our institutional database. The institutional ethics committee approved the protocol, and informed consent was waived due to the retrospective nature of the study (Reference No. 25349; date of approval: January 5, 2026).

## Results

### Baseline Characteristics

The baseline characteristics of patients are summarized in Table 1. The nonsevere iLAD group was significantly younger than the severe iLAD group (67 ± 10 vs 69 ± 9 years, *P* = .002) and had a higher prevalence of left main disease (54.7% vs 44.5%, *P* = .035). In the nonsevere iLAD group, 29.8% of patients had severe stenosis in the pLAD (upstream of the nonsevere iLAD). Regarding the diagonal artery, stenosis severity did not differ between groups (*P* > .05), whereas the sequential graft configuration was more frequently used in the severe iLAD group (66.0% vs 53.4%, *P* = .008). Left ventricular ejection fraction was significantly higher in the nonsevere iLAD group (57% ± 14% vs 54% ± 15%, *P* = .040). Sex, diabetes, or history of myocardial infarction did not show significant differences between the groups.

**Table 1:** Baseline characteristics

Characteristic	Total (N = 496)	Nonsevere iLAD (N = 161)	Severe iLAD (N = 335)	*P* value
Age (y)	69 ± 10	67 ± 10	69 ± 9	**.002**
Male sex	408 (82.3)	131 (81.4)	277 (82.7)	.719
BMI (kg/m^2^)	23.5 ± 3.4	23.7 ± 3.2	23.5 ± 3.5	.432
Current smoker	77 (15.5)	31 (19.3)	46 (13.7)	.114
Hypertension	357 (72.0)	120 (74.5)	237 (70.6)	.395
Diabetes mellitus	279 (56.3)	86 (53.4)	193 (57.6)	.386
Dyslipidemia	343 (69.2)	114 (70.8)	229 (68.4)	.605
eGFR (mL/min/1.73 m^2^)	58.9 ± 23.9	60.8 ± 26.4	58.0 ± 22.6	.226
History of AMI	77 (15.5)	29 (18.0)	48 (14.3)	.292
3-vessel disease	380 (76.6)	119 (73.9)	261 (77.9)	.365
Left main disease	237 (47.8)	88 (54.7)	149 (44.5)	**.035**
LAD stenosis				
<50%	19 (4.0)	19 (11.8)	0	**<.001**
50-69%	3 (0.6)	3 (1.9)	0	**.034**
70-89%	88 (18.3)	91 (56.5)	0	**<.001**
≥90%	370 (77.1)	48 (29.8)	335 (100)	**<.001**
Diagonal stenosis				
<50%	32 (6.5)	12 (7.5)	20 (6.0)	.560
50-69%	4 (0.8)	0	4 (1.2)	.310
70-89%	101 (20.4)	41 (25.5)	60 (17.9)	.057
≥90%	359 (72.4)	108 (67.1)	251 (74.9)	.069
Diagonal graft configuration				
Individual	189 (38.1)	75 (46.6)	114 (34.0)	**.008**
Sequential	307 (61.9)	86 (53.4)	221 (66.0)	**.008**
Concomitant LCX bypass	342 (69.0)	113 (70.2)	229 (68.4)	.756
History of CVD	58 (11.7)	12 (7.5)	46 (13.7)	.052
COPD (moderate or greater)	13 (2.6)	3 (1.9)	10 (3.0)	.562
Peripheral vascular disease	74 (14.9)	28 (17.4)	46 (13.7)	.285
Left ventricular ejection fraction (%)	55 ± 15	57 ± 14	54 ± 15	**.040**
<40%	84 (16.9)	22 (13.7)	62 (18.5)	.353
Preoperative IABP	63 (12.7)	20 (12.4)	43 (12.8)	.897
Preoperative VA-ECMO	4 (0.8)	2 (1.2)	2 (0.6)	.653
Surgical acuity				
Elective	373 (75.2)	121 (75.2)	252 (75.2)	.987
Urgent	52 (10.5)	19 (11.8)	33 (9.9)	.533
Emergency	71 (14.3)	21 (13.0)	50 (14.9)	.682

Values are presented as n (%) or mean ± SD. Bold values indicate statistical significance (*P* < .05). *iLAD*, Intervening left anterior descending; *BMI*, body mass index; *eGFR*, estimated glomerular filtration rate; *AMI*, acute myocardial infarction; *LAD*, left anterior descending; *LCX*, left circumflex artery; *CVD*, cerebrovascular disease; *COPD*, chronic obstructive pulmonary disease; *IABP*, intra-aortic balloon pump; *VA-ECMO*, venoarterial extracorporeal membrane oxygenation.

### Operative Outcomes

**Figure E2:**
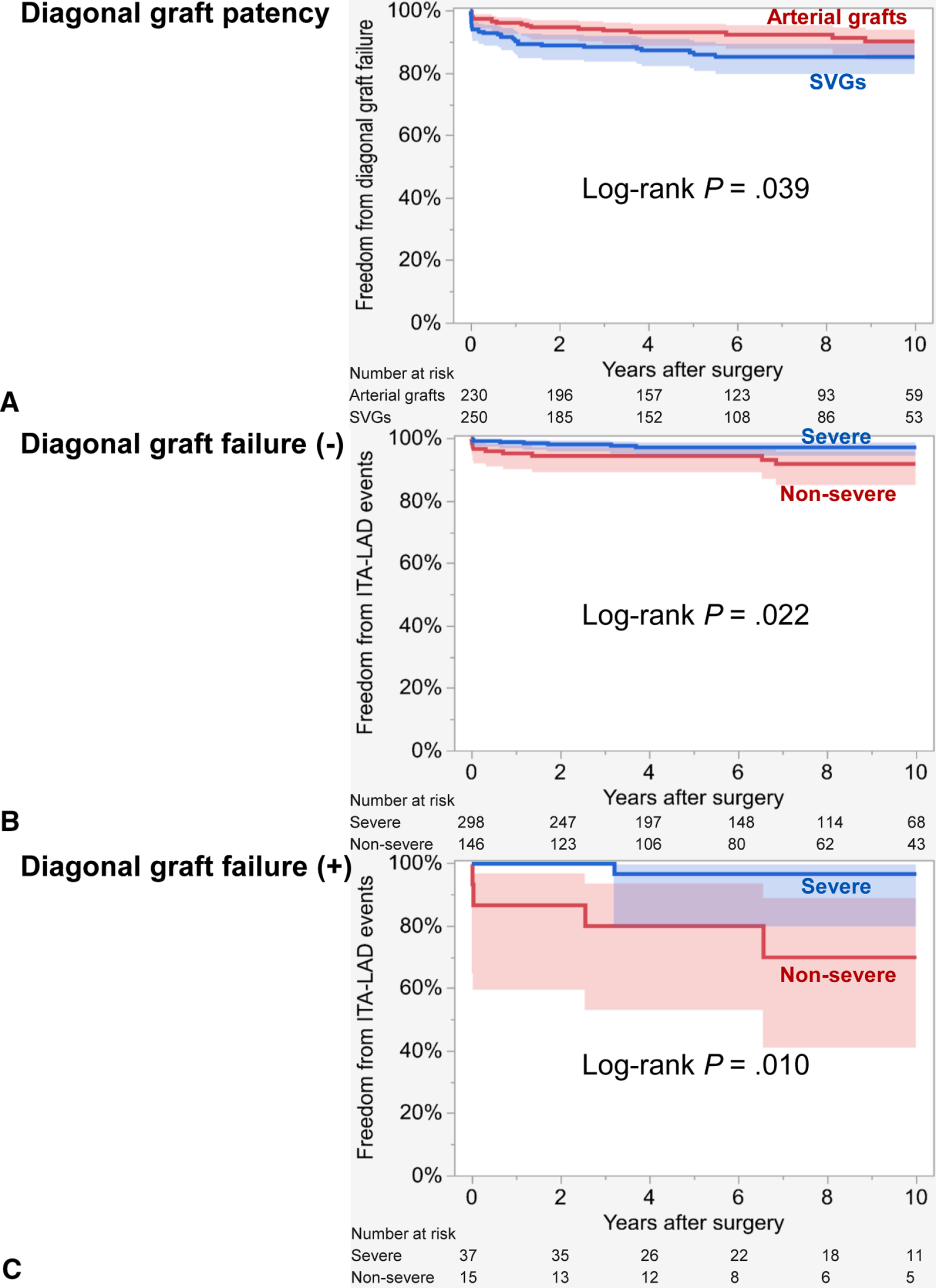
Diagonal graft patency and sensitivity analyses. A, Diagonal graft patency by conduit (arterial grafts vs SVGs). B and C, Freedom from ITA-LAD events by iLAD stenosis severity in patients (B) without and (C) with diagonal graft failure. *iLAD*, Intervening left anterior descending artery; *ITA*, internal thoracic artery; *LAD*, left anterior descending artery; *SVG*, saphenous vein graft.

Table 2 summarizes the operative details and clinical outcomes. There were no significant differences in the total number of anastomoses (3.7 ± 0.9 vs 3.7 ± 0.9, *P* = .991) or the rate of off-pump surgery (73.3% vs 70.5%, *P* = .523) between the nonsevere and severe iLAD groups. There was no significant difference in ITA-LAD graft flow between the nonsevere and severe iLAD groups (37 ± 29 mL/min vs 40 ± 25 mL/min; *P* = .162). In contrast, diagonal graft flow was significantly higher in the severe iLAD group (44 ± 30 mL/min vs 38 ± 26 mL/min; *P* = .049). The 1-year and 5-year diagonal graft patency rates were 96.1% and 93.0% for arterial grafts and 90.6% and 85.9% for SVGs, respectively, which are consistent with reported benchmarks. Sensitivity analyses confirmed that nonsevere iLAD stenosis remained a significant risk factor for ITA-LAD events regardless of diagonal graft failure (without failure, log-rank *P* = .021; with failure, log-rank *P* = .010), demonstrating that this adverse impact is robust and not confounded by early diagonal graft failure (Figure E2).

**Table 2:** Operative outcomes

Variable	Total (N = 496)	Nonsevere iLAD (N = 161)	Severe iLAD (N = 335)	*P* value
Operation time (min)	330 ± 84	334 ± 82	328 ± 84	.397
Cardiopulmonary bypass status				
Off-pump	354 (71.4)	118 (73.3)	236 (70.5)	.523
On-pump beating	140 (28.2)	42 (26.1)	98 (29.3)	.523
Cardiac arrest	2 (0.4)	1 (0.6)	1 (0.3)	.544
No. of anastomosis	3.7 ± 0.9	3.7 ± 0.9	3.7 ± 0.9	.991
Intraoperative transit-time flow				
ITA-LAD graft				
Mean graft flow (mL/min)	39 ± 26	37 ± 29	40 ± 25	.162
Pulsatility index	2.9 ± 1.4	3.1 ± 1.6	2.9 ± 1.3	.165
Diastolic filling (%)	72 ± 12	70 ± 12	74 ± 11	**<.001**
Diagonal graft				
Mean graft flow (mL/min)	42 ± 29	38 ± 26	44 ± 30	**.049**
Pulsatility index	2.7 ± 1.4	2.7 ± 1.3	2.7 ± 1.5	.804
Diastolic filling (%)	70 ± 8	70 ± 7	69 ± 8	.666
Perioperative outcomes				
Operative mortality	2 (0.4)	0	2 (0.6)	1.000
Myocardial infarction	0	0	0	N/A
Cerebrovascular accidents	5 (1.0)	1 (0.6)	4 (1.2)	1.000
Long-term outcomes				
Follow-up period (y)	6.8 [3.6-10.3]	7.3 [4.2-11.3]	6.6 [3.2-9.9]	**.047**
Mortality	150 (30.2)	47 (29.2)	103 (30.8)	.331
Cardiac death	46 (9.3)	15 (9.3)	31 (9.3)	.712
Myocardial infarction	7 (1.4)	4 (2.5)	3 (0.9)	.215
Cerebrovascular accidents	34 (6.9)	9 (5.6)	25 (7.5)	.302
Reintervention	51 (10.3)	19 (11.8)	32 (9.6)	.527
ITA-LAD events	22 (4.4)	14 (8.7)	8 (2.4)	**.002**
Myocardial infarction	1 (0.2)	1 (0.62)	0	.145
Reintervention	6 (1.2)	3 (1.9)	3 (0.9)	.387
Occlusion	7 (1.4)	5 (3.1)	2 (0.6)	**.029**
String sign or stenosis	8 (1.6)	5 (3.1)	3 (0.9)	.074

Values are presented as n (%), mean ± SD, or median [interquartile range]. Bold values indicate statistical significance (*P* < .05). *P* values for long-term outcomes (excluding follow-up period) were calculated using the log-rank test. Other *P* values were calculated using Fisher exact test or the Mann–Whitney *U* test. *iLAD*, Intervening left anterior descending; *ITA*, internal thoracic artery; *LAD*, left anterior descending; *N/A*, not applicable.

With regard to perioperative outcomes, operative mortality (0% and 0.6% in the nonsevere and severe iLAD groups, respectively; *P* > .999) and incidence of perioperative stroke (0.6% and 1.2% in the nonsevere and severe groups, respectively; *P* > .999) were not statistically different between the nonsevere and severe groups. Perioperative myocardial infarction was not observed in either group.

**Figure E3:**
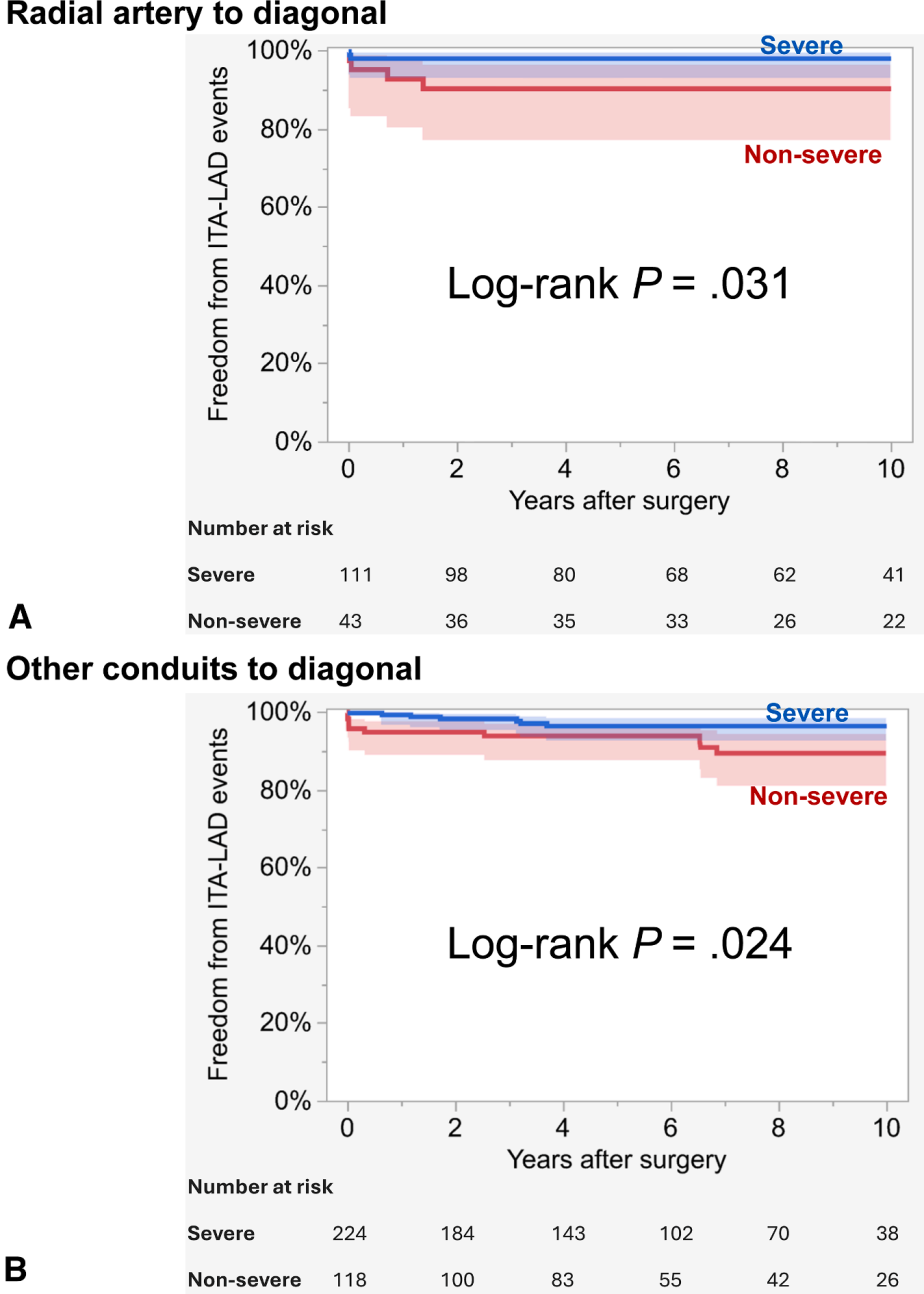
Sensitivity analysis by radial artery use. Kaplan–Meier analyses showing freedom from ITA-LAD events stratified by iLAD stenosis severity within the (A) with radial artery subgroup (n = 154) and (B) without radial artery subgroup (n = 342). *P* values were calculated using the log-rank test. CI = 95%. *iLAD*, Intervening left anterior descending artery; *ITA*, internal thoracic artery; *LAD*, left anterior descending artery.

The median follow-up period was 6.8 years (IQR, 3.6-10.3 years). The nonsevere iLAD group had a significantly longer follow-up period compared with the severe iLAD group (7.3 [4.2-11.3] years vs 6.6 [3.2-9.9] years; *P* = .047). During follow-up, there were no significant differences in all-cause mortality or cardiac death (log-rank *P* = .755, .576, respectively) between the groups. Additionally, the rates of myocardial infarction, cerebrovascular accidents, and coronary reintervention (log-rank *P* = .221, .570, .434, respectively) were also comparable. However, ITA-LAD events were significantly more frequent in the nonsevere iLAD group than in the severe iLAD group (8.7% vs 2.4%; log-rank *P* = .002). Specifically, the incidence of ITA-LAD occlusion was significantly higher in the nonsevere iLAD group than in the severe iLAD group (3.1% vs 0.6%; log-rank *P* = .015). Furthermore, an exploratory sensitivity analysis stratified by radial artery use to the diagonal branch (radial artery, n = 154; other conduits, n = 342) revealed no overall difference in freedom from ITA-LAD events (log-rank *P* = .670). The association between nonsevere intervening stenosis and an increased risk of graft events remained consistent among both patients without radial artery use (log-rank *P* = .024) and those with radial artery use (log-rank *P* = .031) (Figure E3).

### Subgroup Analysis Stratified by Stenosis Severity in Three Distinct Segments

**Figure E4:**
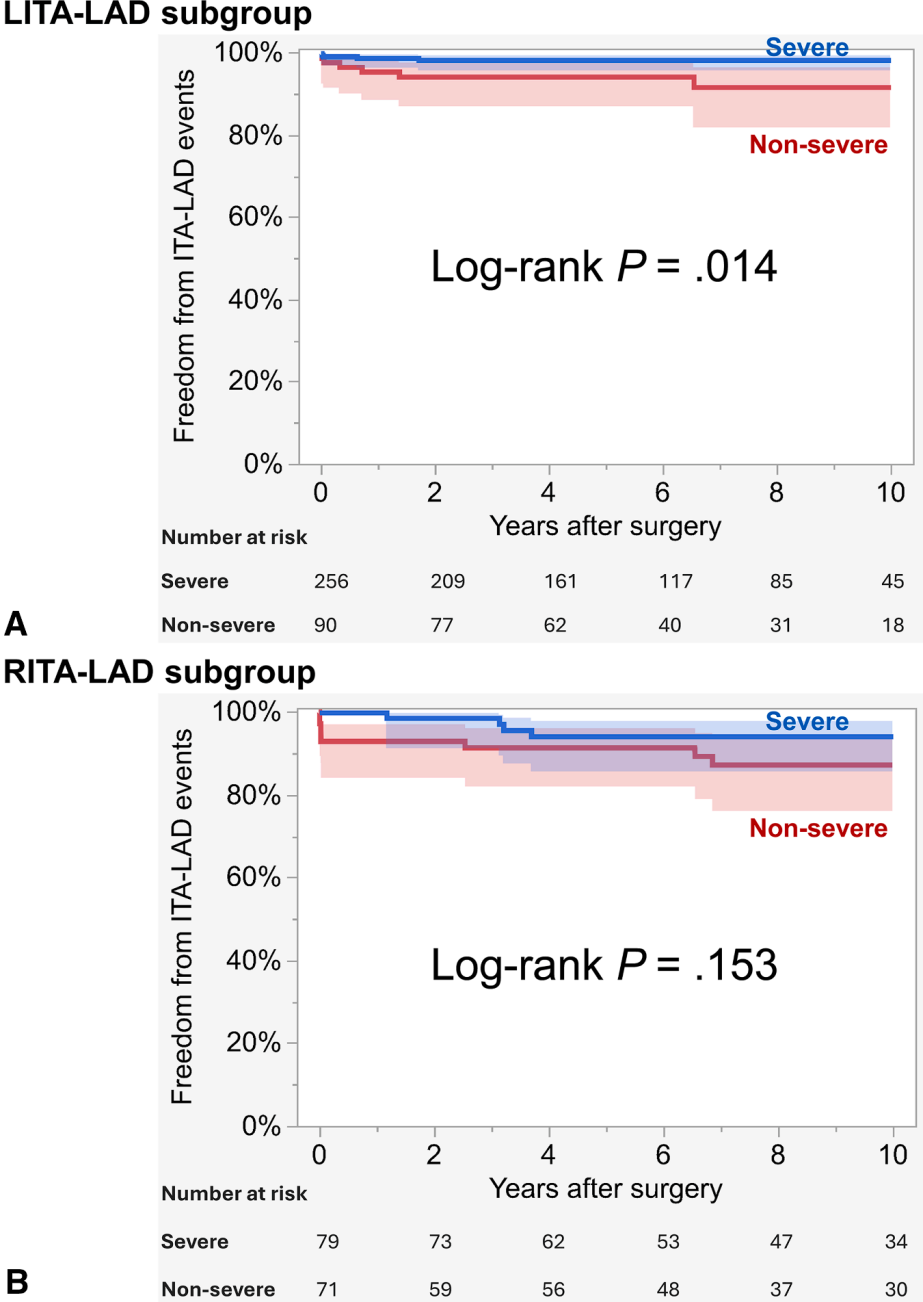
Sensitivity analysis by internal thoracic artery side. Kaplan–Meier analyses showing freedom from ITA-LAD events stratified by iLAD stenosis severity within the (A) LITA-LAD subgroup (n = 346) and (B) RITA-LAD subgroup (n = 150). *P* values were calculated using the log-rank test. CI = 95%. *iLAD*, Intervening left anterior descending artery; *ITA*, internal thoracic artery; *LAD*, left anterior descending artery; *LITA*, left internal thoracic artery; *RITA*, right internal thoracic artery.

Kaplan–Meier analyses stratified by stenosis severity in 3 distinct segments are shown in Figure 2. In the iLAD segment, the nonsevere iLAD group demonstrated significantly lower freedom from ITA-LAD events than the severe iLAD group (log-rank *P* = .002). Freedom from ITA-LAD events at 1, 5, and 10 years in the nonsevere and severe iLAD groups was 94.3% ± 1.8%, 92.9% ± 2.1%, and 89.5% ± 2.8% versus 99.1% ± 0.5%, 97.2% ± 1.0%, and 97.2% ± 1.0%, respectively. Regarding the diagonal artery and pLAD, there were no significant differences in freedom from ITA-LAD events stratified by stenosis severity (log-rank *P* = .391, .399, respectively). Additionally, sensitivity analysis stratified by the ITA side (left ITA, n = 346; right ITA, n = 150; Figure E4) demonstrated that freedom from ITA-LAD events was significantly lower in the nonsevere iLAD group within the left ITA-LAD subgroup (log-rank *P* = .014), whereas the right ITA-LAD subgroup showed a similar trend but did not reach statistical significance (log-rank *P* = .153), which likely represents a type II error due to the limited sample size.

**Figure 1:**
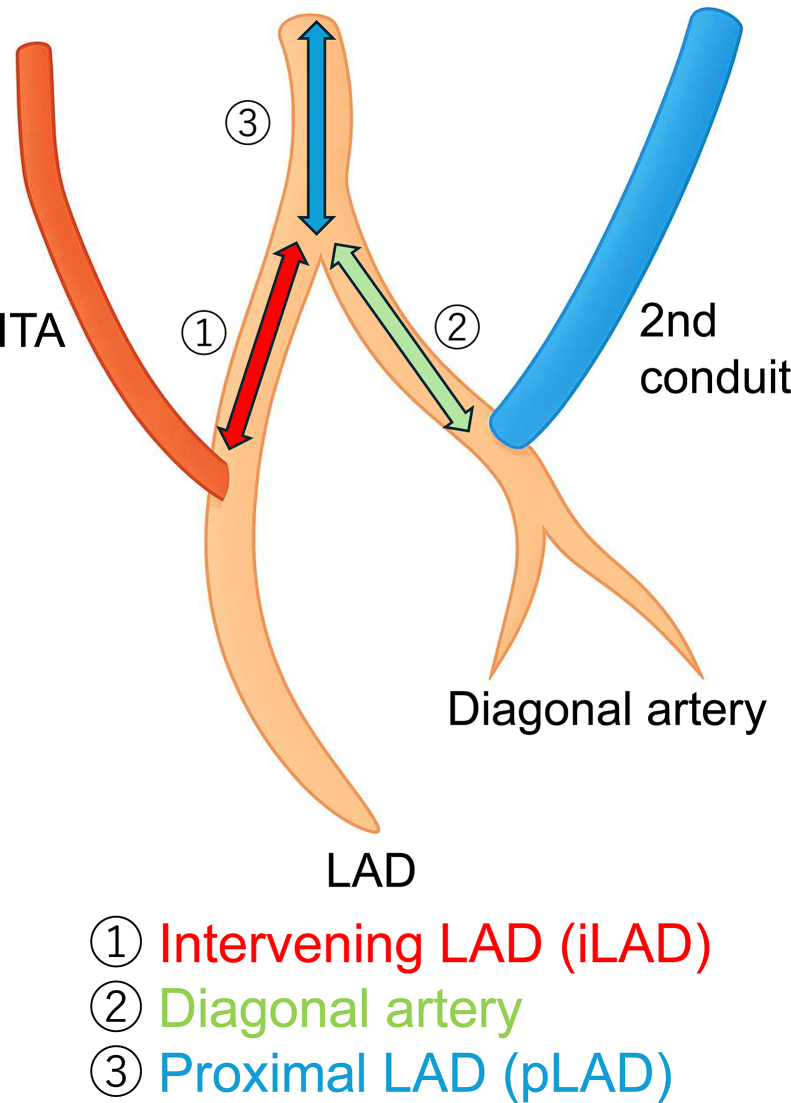
Relevant coronary segments. Stenosis severity was evaluated in 3 specific segments defined relative to the target bypassed diagonal artery (closest to the ITA-LAD anastomosis): ① iLAD: The LAD segment between the ITA-LAD anastomosis and the diagonal artery origin. ② Diagonal artery: The target diagonal artery itself. ③ pLAD: The LAD segment proximal to the diagonal artery origin, including the left main trunk. *iLAD*, Intervening left anterior descending artery; *ITA*, internal thoracic artery; *LAD*, left anterior descending artery; *pLAD*, proximal left anterior descending artery.

**Figure 2:**
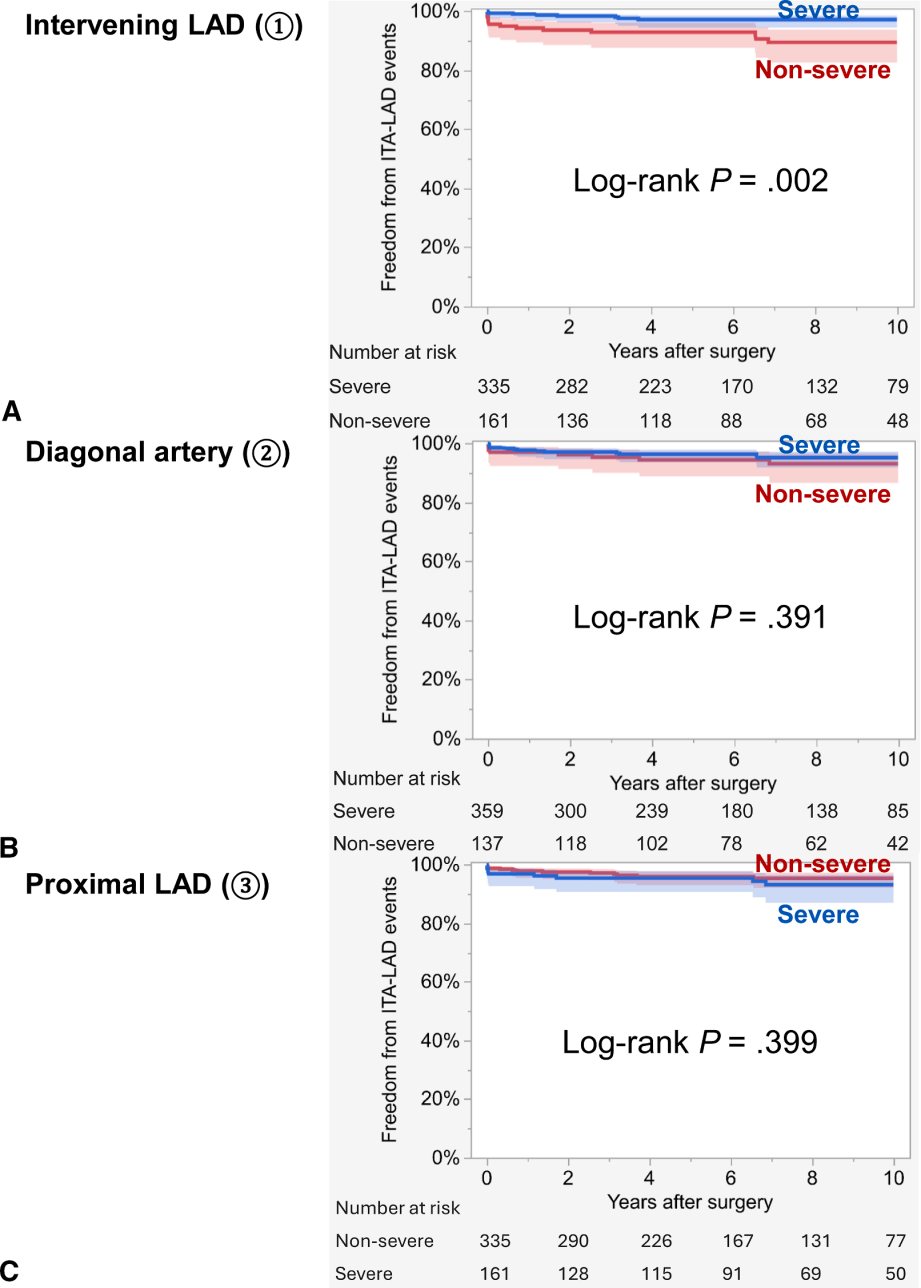
Kaplan–Meier analysis of freedom from ITA-LAD events stratified by stenosis severity in 3 distinct segments. The curves show the freedom from ITA-LAD events for the entire cohort, stratified by the stenosis severity of (A) the iLAD (①), (B) the diagonal artery (②), and (C) the pLAD (③), as defined in Figure 1. The shaded areas indicate 95% CI. *ITA*, Internal thoracic artery; *LAD*, left anterior descending.

### Impact of Adjacent Vessel Stenosis

**Figure 3:**
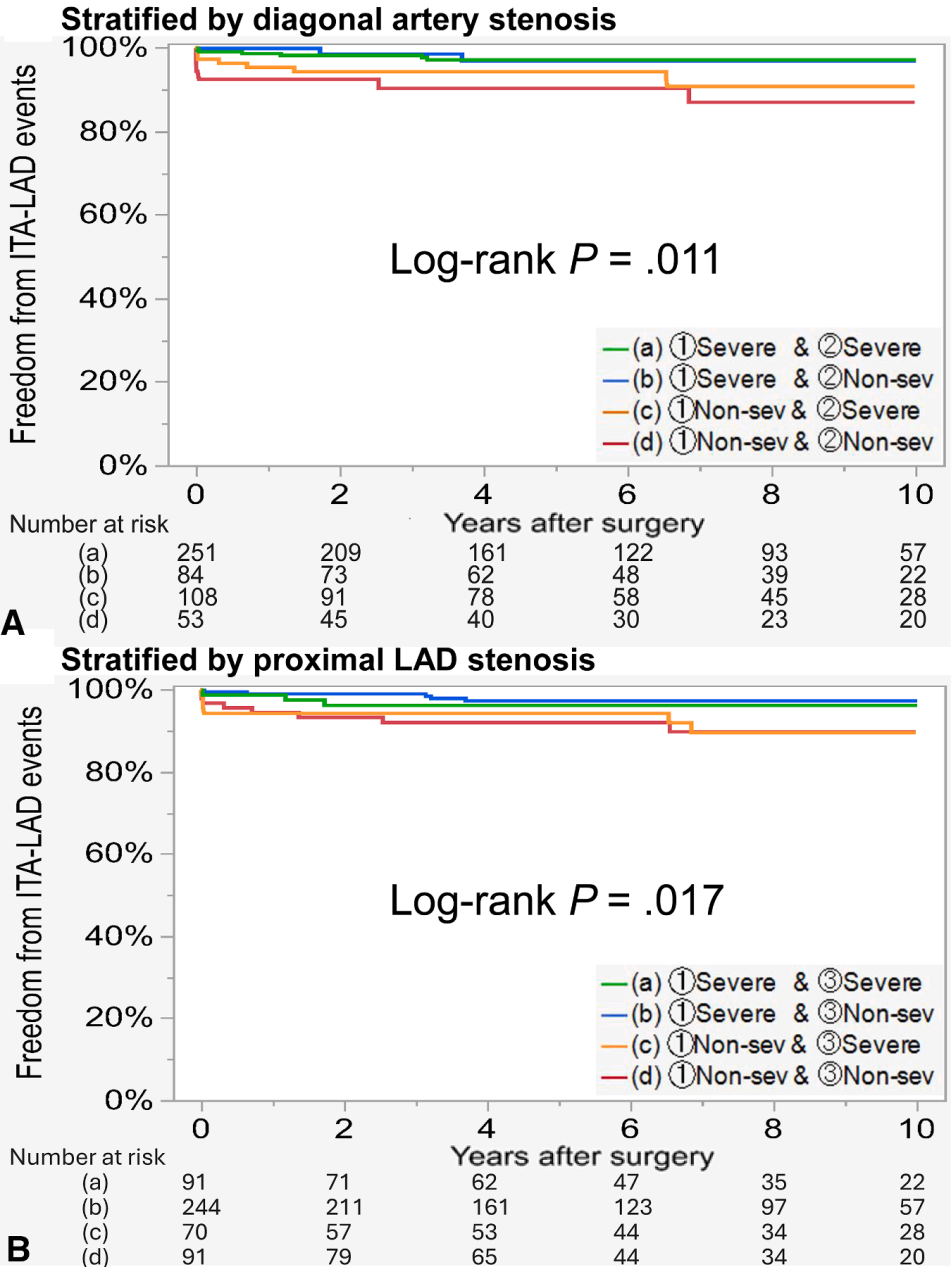
Impact of iLAD stenosis on ITA-LAD graft events stratified by adjacent vessel stenosis. The curves show the freedom from ITA-LAD events for 4 subgroups defined by the combination of iLAD (①) stenosis severity and that of (A) the diagonal artery (②) or (B) the pLAD (③) as defined in Figure 1. The point estimates and 95% CI are presented in Table E1. *ITA*, Internal thoracic artery; *LAD*, left anterior descending.

In the pLAD stenosis analysis, freedom from ITA-LAD events was significantly lower in patients with nonsevere iLAD, regardless of the extent of pLAD stenosis. Even with severe pLAD stenosis, nonsevere iLAD was associated with poorer outcomes than in the severe iLAD group (log-rank *P* = .011). In the diagonal stenosis analysis, subgroups with nonsevere iLAD stenosis demonstrated consistently lower freedom from events, irrespective of the diagonal stenosis severity (log-rank *P* = .017) (Figure 3 and Table E1).

### Impact of Diagonal Graft Configuration

**Figure 4:**
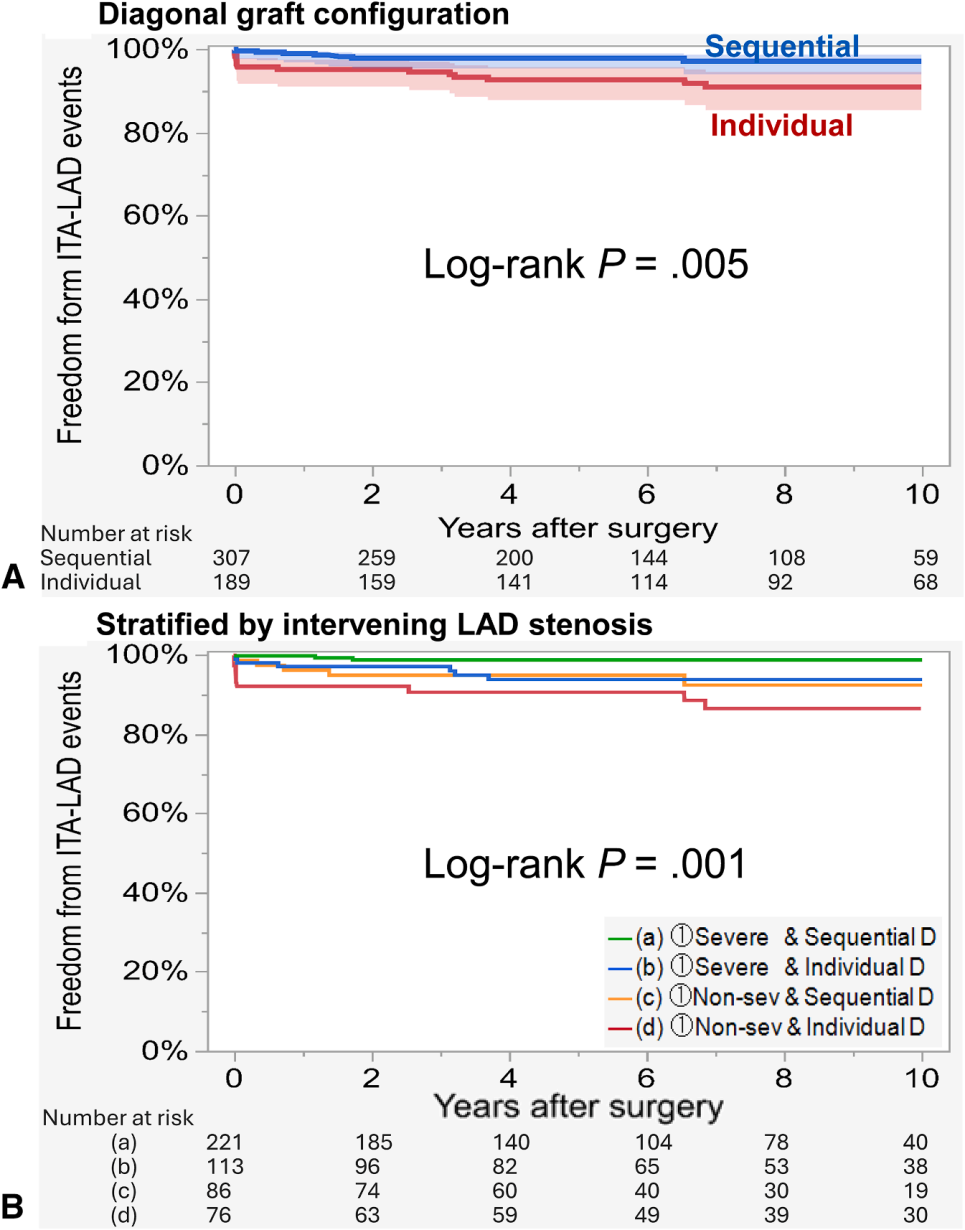
Impact of diagonal graft configuration and its combined effect with iLAD stenosis on ITA-LAD events. Kaplan–Meier analyses showing freedom from ITA-LAD events stratified by (A) diagonal graft configuration (individual vs sequential) and (B) the combination of iLAD (①) stenosis severity, as defined in Figure 1, and diagonal graft configuration. The shaded areas in (A) indicate 95% CI. The point estimates and 95% CI for (B) are listed in Table E1. *D*, Diagonal artery; *ITA*, internal thoracic artery; *LAD*, left anterior descending.

The individual diagonal graft configuration was significantly associated with a lower freedom from ITA-LAD events than the sequential diagonal graft configuration (log-rank *P* = .005). The freedom from ITA-LAD events at 1, 5, and 10 years was 95.2% ± 1.6%, 92.7% ± 2.0%, and 91.0% ± 2.3% in the individual graft group, respectively, compared with 99.0% ± 0.6%, 97.9% ± 0.8%, and 97.2% ± 1.1% in the sequential graft group, respectively. Regarding interactions with iLAD stenosis, the combination of severe iLAD stenosis and sequential diagonal bypass resulted in the most favorable outcomes. In contrast, the combination of nonsevere iLAD stenosis and individual diagonal bypass was associated with the poorest freedom from ITA-LAD events (log-rank *P* = .001) (Figure 4 and Table E1).

### Predictors of Internal Thoracic Artery–Left Anterior Descending Events

Multivariate Cox proportional hazards analysis identified nonsevere iLAD (adjusted HR, 2.59; 95% CI, 1.07-6.30; *P* = .035) and individual diagonal graft configuration (adjusted HR, 2.57; 95% CI, 1.04-6.36; *P* = .041) as independent risk factors for ITA-LAD events (Table 3). Conversely, higher intraoperative ITA-LAD graft flow (adjusted HR, 0.94; 95% CI, 0.90-0.98; *P* = .006) was identified as a significant independent predictor of a lower risk of ITA-LAD events. Other stenosis factors, including diagonal stenosis, pLAD stenosis, and concomitant left circumflex artery grafting, were not significant predictors.

**Table 3:** Risk analysis for internal thoracic artery left anterior descending events: Cox proportional hazards regression analysis

Variable	Univariate analysis		Multivariate analysis	
	Crude HR (95% CI)	*P* value	Adjusted HR (95% CI)	*P* value
iLAD stenosis [Nonsevere]	3.67 (1.54-8.76)	**.003**	2.59 (1.07-6.30)	**.035**
Diagonal stenosis [Nonsevere]	1.46 (0.61-3.48)	.393		
pLAD stenosis [Nonsevere]	0.70 (0.30-1.63)	.402		
Diagonal graft configuration [Individual]	3.35 (1.36-8.22)	**.008**	2.57 (1.04-6.36)	**.041**
Age	1.00 (0.96-1.05)	.953	1.00 (0.96-1.05)	.860
Female sex	3.23 (1.38-7.56)	**.007**	2.04 (0.85-4.90)	.109
BMI (kg/m^2^)	1.07 (0.95-1.19)	.253		
Hypertension	1.30 (0.48-3.54)	.601		
Diabetes mellitus	0.70 (0.30-1.62)	.402		
Dyslipidemia	1.15 (0.45-2.93)	.773		
eGFR (mL/min/1.73 m^2^)	1.00 (0.98-1.02)	.961		
Prior acute myocardial infarction	0.53 (0.12-2.27)	.394		
3-vessel disease	0.68 (0.28-1.66)	.394		
Left main disease	0.86 (0.37-2.00)	.733		
Concomitant LCX grafting	0.73 (0.31-1.74)	.481		
History of CVD	0.78 (0.18-3.35)	.742		
Peripheral vascular disease	0.60 (0.14-2.56)	.490		
Left ventricular ejection fraction (%)	1.02 (0.99-1.06)	.134		
Low EF (<40%)	0.51 (0.12-2.17)	.657		
Elective	0.84 (0.33-2.16)	.725		
Urgent	1.99 (0.67-5.89)	.212		
Emergency	0.61 (0.14-2.62)	.509		
No. of anastomosis	0.76 (0.48-1.22)	.257		
Operation time	1.00 (1.00-1.01)	.585		
Off-pump	2.45 (0.72-8.27)	.150		
ITA-LAD graft flow	0.93 (0.88-0.96)	**.001**	0.94 (0.90-0.98)	**.006**
Diagonal graft flow	0.99 (0.97-1.01)	.283		

Bold values indicate statistical significance (*P* < .05). *HR*, Hazard ratio; *iLAD*, intervening left anterior descending artery; *pLAD*, proximal left anterior descending artery; *LAD*, left anterior descending; *BMI*, body mass index; *eGFR*, estimated glomerular filtration rate; *LCX*, left circumflex; *CVD*, cardiovascular disease; *EF*, ejection fraction; *ITA-LAD*, internal thoracic artery left anterior descending.

## Discussion

This study investigated the hemodynamic interaction between multi-segmental stenosis severity and graft configuration in combined ITA-LAD and diagonal bypass surgery and found (1) nonsevere iLAD stenosis was an independent risk factor for ITA-LAD events; (2) pLAD and diagonal stenosis severity did not affect the primary outcome; (3) sequential diagonal grafting mitigated ITA-LAD events risk compared with individual grafting; and (4) higher intraoperative ITA-LAD flow was an independent predictor of reduced ITA-LAD events risk.

The anatomic configuration may carry a risk of competitive flow. However, the precise anatomic determinants remain unclear. In inter-anastomotic stenosis, competitive flow from a secondary graft compromises ITA patency when the native stenosis between anastomoses is nonsevere.^6^ A typical example is observed in patients with left main disease undergoing bypass with ITA-LAD and a second graft to the circumflex artery, where no significant stenosis exists between the 2 anastomotic sites. Specifically for diagonal grafting, computational fluid dynamics simulations show that a single diagonal graft generates systolic reverse flow toward the LAD, which theoretically interferes with antegrade ITA flow.^9^ A case report documenting functional ITA occlusion due to flow competition from a patent SVG-diagonal graft supports this claim.^8^ Our study refines these observations by identifying iLAD stenosis severity as the primary anatomic determinant of ITA-LAD patency. Notably, contrary to simulation models suggesting that mild diagonal stenosis maximizes the reverse flow risk, our data show that diagonal stenosis severity was not associated with ITA-LAD events, suggesting iLAD stenosis is the dominant anatomic factor that determines outcomes.

Its decisive role can be explained by the balance between native inflow and the graft. Physiologically, the total blood flow required in the distal LAD territory is primarily determined by myocardial oxygen demand (runoff).^10^^,^^11^ Under constant demand, the proportion of flow supplied by the ITA graft depends on iLAD stenosis. Severe stenosis imposes high resistance and a significant pressure drop, compelling the ITA to supply the majority of blood flow to the area. This high-flow state is essential for physiological shear stress and endothelial function, protecting the graft.^10^^,^^11^ Conversely, nonsevere stenosis allows native flow to maintain high pressure at the anastomotic site. This impedes ITA inflow and predisposes it to atrophy or failure.^8^^,^^9^

The finding that diagonal severity did not affect event rates contrasts with computational fluid dynamics simulations, which predicted maximum reverse flow risk with mild stenosis.^9^ Although nonsevere diagonal stenosis likely increased reverse flow volume, our data suggest that such increases are insufficient to affect clinical outcomes. This seeming inconsistency reflects a clear hierarchy of anatomic factors regulating competitive flow. Instead, iLAD stenosis acted as the dominant regulator of competitive flow reaching the anastomosis. The competitive blood flow threatening the ITA-LAD graft corresponded to the total flow passing through the iLAD segment. Although the stenosis severity of the diagonal or pLAD arteries may alter the volume or source of this native inflow (comprising both pLAD forward and diagonal graft reverse flows), the net impact on the ITA graft is regulated by the resistance of the iLAD stenosis itself. Consequently, upstream factors, including diagonal and pLAD stenoses, were effectively filtered by iLAD stenosis.

Although individual diagonal grafting was identified as an independent risk factor of ITA-LAD, sequential grafting mitigated this risk. It introduces a high-pressure systemic source without an alternative outlet, augmenting the retrograde component of the native inflow and exerting maximal competitive stress on the ITA.^11^ In contrast, sequential grafting extends to additional territories (eg, the circumflex or right coronary arteries), providing distal runoff to accommodate flow. This leaves insufficient driving pressure to sustain the retrograde flow toward the iLAD segment.^12^

**Figure E5:**
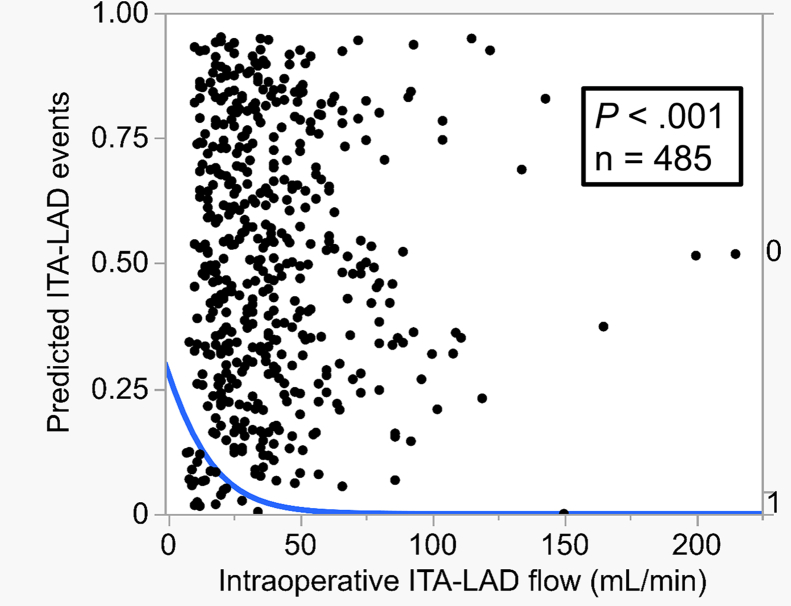
Relationship between intraoperative ITA-LAD graft flow and the probability of graft events. Scatter plot with a fitted logistic regression curve demonstrating the relationship between intraoperative transit-time graft flow and the predicted probability of ITA-LAD events (univariate logistic regression, *P* < .001; n = 485). *ITA*, Internal thoracic artery; *LAD*, left anterior descending artery.

Furthermore, higher intraoperative ITA-LAD flow independently predicted reduced ITA-LAD events risk, aligning with evidence validating quantitative flow assessment prognostic utility.^13^ High flow integrates stenosis severity, distal runoff, and anastomotic resistance, serving as a metric of demand-supply matching.^10^ Thus, high intraoperative graft flow serves as a practical surrogate for long-term ITA-LAD patency.^13^ However, static intraoperative flow measurement primarily confirms technical anastomotic quality. Crucially, because these assessments are performed when myocardial oxygen demand is at its nadir, they are inherently limited in their ability to unmask the dynamic and variable flow competition that occurs during the postoperative period under physiological stress.^14^ Therefore, the observed differences in late graft failure are physiologically due to the severity of native stenosis rather than initial anastomotic flaws. Although exceptionally high flow can overcome unfavorable hemodynamics (Figure E5), intervening stenosis remained an independent risk factor, particularly within the ordinary flow range. Although complete revascularization is generally recommended and the loss of diagonal branch flow has been linked to adverse outcomes,^15^^,^^16^ these findings highlight the need to carefully weigh the prognostic benefit of a diagonal bypass against the specific risk of flow competition to the ITA graft. Furthermore, we recognize that intraoperative surgical judgment regarding conduit quality, length, and other technical factors inevitably influences the choice of grafting configuration. Because these factors were not systematically captured, they constitute an inherent limitation of our retrospective analysis. However, in our series, the use of a separate conduit for the diagonal branch was predominantly based on an institutional policy to prioritize an independent ITA-LAD bypass. Within this strategy, our findings demonstrated that an individual diagonal graft is a risk factor for ITA-LAD events when iLAD stenosis is nonsevere. This suggests that a sequential diagonal graft, extending to other territories to facilitate run-off, might be a superior alternative to mitigate competitive flow toward the LAD. Nevertheless, an individual diagonal graft can be justified even in such cases, as long as the ITA-LAD graft maintains sufficient flow capacity to overcome potential competition (Figure E5). Establishing precise indications for diagonal grafting remains a crucial subject for future investigation.

### Limitations

This was a single-center retrospective study with potential selection bias. Stenosis severity was assessed using angiography without physiological data (eg, fractional flow reserve),^17^ and transit-time flowmetry values for sequential grafts reflect cumulative flow, preventing isolation of specific diagonal component.^7^ Furthermore, postoperative imaging was not universal; because late angiography was clinically driven rather than a routine follow-up, silent ITA-LAD string signs might not have been captured, thus potentially underestimating asymptomatic graft failures. Finally, the absolute number of clinical events was small. This limits the statistical power and robustness of our conclusions, and the results should be interpreted with caution.

## Conclusions

iLAD stenosis is a key determinant of ITA-LAD outcomes, independent of pLAD or diagonal disease severity. Sequential diagonal grafting mitigates the excess risk associated with nonsevere iLAD stenosis, supporting the importance of grafting strategies tailored to iLAD lesion characteristics to ensure durable ITA-LAD patency.

### Webcast

You can watch a Webcast of this AATS meeting presentation by going to: https://www.aats.org/resources/influence-of-the-severity-of-s-12227.

**Figure:**
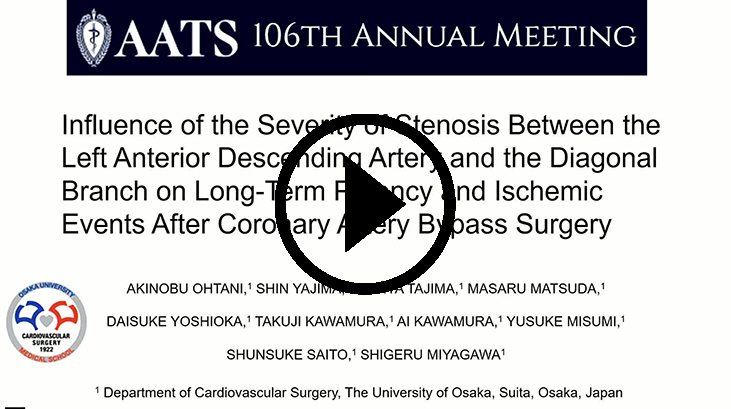

## Conflict of Interest Statement

The authors reported no conflicts of interest.

The *Journal* policy requires editors and reviewers to disclose conflicts of interest and to decline handling or reviewing manuscripts for which they may have a conflict of interest. The editors and reviewers of this article have no conflicts of interest.

## Figures and Tables

**Table E1:** Point estimates and 95% CI for freedom from internal thoracic artery left anterior descending events shown in Figure 3, *A* and *B*, and Figure 4, *B*

Group	1 y (95% CI)	5 y (95% CI)	10 y (95% CI)
Figure 3, *A*: Stratified by diagonal stenosis			
(a) ①Severe and ②severe	98.9 (92.6-99.8)	96.4 (89.3-98.8)	96.4 (89.3-98.8)
(b) ①Severe and ②nonsevere	99.2 (96.7-99.8)	97.5 (94.0-99.0)	97.5 (94.0-99.0)
(c) ①Nonsevere and ②severe	94.2 (85.6-97.8)	94.2 (85.6-97.8)	89.5 (78.0-95.4)
(d) ①Nonsevere and ②nonsevere	94.4 (87.3-97.7)	92.0 (84.1-96.1)	89.7 (80.3-95.0)
Figure 3, *B*: Stratified by pLAD stenosis			
(a) ①Severe and ③severe	98.8 (96.3-99.6)	97.3 (94.0-98.8)	97.3 (94.0-98.8)
(b) ①Severe and ③nonsevere	100	97.1 (89.0-99.3)	97.1 (89.0-99.3)
(c) ①Nonsevere and ③severe	95.3 (89.2-98.0)	94.3 (87.8-97.4)	90.7 (81.9-95.5)
(d) ①Nonsevere and ③nonsevere	92.4 (81.5-97.1)	90.3 (78.6-95.9)	86.9 (73.2-94.2)
Figure 4, *B*: Stratified by iLAD stenosis			
(a) ①Severe and sequential D	100	99.0 (96.0-99.7)	99.0 (96.0-99.7)
(b) ①Severe and individual D	97.3 (92.0-99.1)	94.1 (87.4-97.3)	94.1 (87.4-97.3)
(c) ①Nonsevere and sequential D	96.4 (89.5-98.8)	95.1 (87.8-98.2)	92.6 (82.6-97.1)
(d) ①Nonsevere and individual D	92.1 (83.5-96.4)	90.6 (81.6-95.5)	86.5 (75.7-93.0)

① iLAD, ② diagonal artery, and ③ pLAD. *pLAD*, Proximal left anterior descending; *iLAD*, intervening left anterior descending artery; *D*, diagonal artery.

## References

[1] Lawton J.S., Tamis-Holland J.E., Bangalore S. (2021). ACC/AHA/SCAI guideline for coronary artery revascularization: a report of the American College of Cardiology/American Heart Association joint committee on clinical practice guidelines. Circulation.

[2] Vrints C., Andreotti F., Koskinas K.C. (2024). ESC guidelines for the management of chronic coronary syndromes. Eur Heart J.

[3] Sabik J.F., Lytle B.W., Blackstone E.H., Khan M., Houghtaling P.L., Cosgrove D.M. (2003). Does competitive flow reduce internal thoracic artery graft patency?. Ann Thorac Surg.

[4] Kim M.S., Ryu A.J., Kim J.W., Lee C.H., Hwang S.W., Kim K.B. (2026). Prediction of graft patency during the year following coronary artery bypass grafting: preoperative computed tomography-derived fractional flow reserve versus intraoperative transit-time flow measurement. J Thorac Cardiovasc Surg.

[5] Harskamp R.E., Alexander J.H., Ferguson T.B. (2016). Frequency and predictors of internal mammary artery graft failure and subsequent clinical outcomes: insights from the Project of Ex-vivo Vein Graft Engineering via Transfection (PREVENT) IV trial. Circulation.

[6] Kawamura M., Nakajima H., Kobayashi J. (2008). Patency rate of the internal thoracic artery to the left anterior descending artery bypass is reduced by competitive flow from the concomitant saphenous vein graft in the left coronary artery. Eur J Cardiothorac Surg.

[7] Akhrass R., Bakaeen F.G. (2021). Intraoperative graft patency validation: friend or foe?. JTCVS Tech.

[8] Papakonstantinou K., Lerios P., Argiriou M. (2025). Should we ligate coronary conduits when flow measurements indicate competitive flow? A case report. J Cardiothorac Surg.

[9] Koyama S., Itatani K., Yamamoto T. (2014). Optimal bypass graft design for left anterior descending and diagonal territory in multivessel coronary disease. Interact Cardiovasc Thorac Surg.

[10] Pagni S., Storey J., Ballen J. (1997). ITA versus SVG: a comparison of instantaneous pressure and flow dynamics during competitive flow. Eur J Cardiothorac Surg.

[11] Meng X., Fu Q., Sun W., Yu J., Yue W., Bi Y. (2013). Competitive flow arising from varying degrees of coronary artery stenosis affects the blood flow and the production of nitric oxide and endothelin in the internal mammary artery graft. Eur J Cardiothorac Surg.

[12] Taggart D.P., Thuijs D.J.F.M., Di Giammarco G. (2020). Intraoperative transit-time flow measurement and high-frequency ultrasound assessment in coronary artery bypass grafting. J Thorac Cardiovasc Surg.

[13] Kieser T.M., Rose S., Kowalewski R., Belenkie I. (2010). Transit-time flow predicts outcomes in coronary artery bypass graft patients: a series of 1000 consecutive arterial grafts. Eur J Cardiothorac Surg.

[14] Gaudino M., Sandner S., Di Giammarco G. (2021). The use of intraoperative transit time flow measurement for coronary artery bypass surgery: systematic review of the evidence and expert opinion statements. Circulation.

[15] Zhang S., Deng X., Yang W. (2020). The diagonal branches and outcomes in patients with anterior ST-elevation myocardial infarction. BMC Cardiovasc Disord.

[16] Bianco V., Kilic A., Aranda-Michel E. (2023). Complete revascularization during coronary artery bypass grafting is associated with reduced major adverse events. J Thorac Cardiovasc Surg.

[17] Glineur D., Grau J.B., Etienne P.Y. (2019). Impact of preoperative fractional flow reserve on arterial bypass graft anastomotic function: the IMPAG trial. Eur Heart J.

